# Decreased expression of axon-guidance receptors in the anterior cingulate cortex in autism

**DOI:** 10.1186/2040-2392-2-14

**Published:** 2011-08-22

**Authors:** Shiro Suda, Keiko Iwata, Chie Shimmura, Yosuke Kameno, Ayyappan Anitha, Ismail Thanseem, Kazuhiko Nakamura, Hideo Matsuzaki, Kenji J Tsuchiya, Genichi Sugihara, Yasuhide Iwata, Katsuaki Suzuki, Keita Koizumi, Haruhiro Higashida, Nori Takei, Norio Mori

**Affiliations:** 1 Research Center for Child Mental Development, Hamamatsu University School of Medicine, Hamamatsu 431-3192 Japan; 2 Department of Psychiatry & Neurology, Hamamatsu University School of Medicine, Hamamatsu 431-3192 Japan; 3 Research Center for Child Mental Development, Kanazawa University School of Medicine, Kanazawa Japan; 4 Division of Psychological Medicine, Institute of Psychiatry, London SE5 8AF, UK

## Abstract

**Background:**

Axon-guidance proteins play a crucial role in brain development. As the dysfunction of axon-guidance signaling is thought to underlie the microstructural abnormalities of the brain in people with autism, we examined the postmortem brains of people with autism to identify any changes in the expression of axon-guidance proteins.

**Results:**

The mRNA and protein expression of axon-guidance proteins, including ephrin (EFN)A4, eEFNB3, plexin (PLXN)A4, roundabout 2 (ROBO)2 and ROBO3, were examined in the anterior cingulate cortex and primary motor cortex of autistic brains (n = 8 and n = 7, respectively) and control brains (n = 13 and n = 8, respectively) using real-time reverse-transcriptase PCR (RT-PCR) and western blotting. Real-time RT-PCR revealed that the relative expression levels of EFNB3, PLXNA4A and ROBO2 were significantly lower in the autistic group than in the control group. The protein levels of these three genes were further analyzed by western blotting, which showed that the immunoreactive values for PLXNA4 and ROBO2, but not for EFNB3, were significantly reduced in the ACC of the autistic brains compared with control brains.

**Conclusions:**

In this study, we found decreased expression of axon-guidance proteins such as PLXNA4 and ROBO2 in the brains of people with autism, and suggest that dysfunctional axon-guidance protein expression may play an important role in the pathophysiology of autism.

## Introduction

Autism is a pervasive developmental disorder characterized by impairments in reciprocal social interactions and communication, and the presence of repetitive and stereotyped behaviors [1]. Emerging evidence from neuroimaging studies suggests that there are abnormalities of growth, microstructure and neuronal connectivity in the brains of people with autism. For example, using magnetic resonance imaging (MRI) with a diffusion tensor imaging (DTI) technique, Barnea-Goraly *et al. *showed reduced fractional anisotropy (FA), which is indicative of white-matter structural alterations, in the anterior cingulate cortex (ACC) of people with autism, a region known to be related to social cognition [2]. This finding suggests that impaired structural integrity in the white matter may play an important role in the pathophysiology of autism. Because autistic symptoms begin in the first years of life and brain-growth abnormalities precede the onset of these symptoms, it has been suggested that the microstructural changes in the brain persist from the early developmental stage [3].

At the cellular level, the growing neuronal cells during brain development express receptors for families of axon-guidance molecules, such as netrins, slits, ephrins and semaphorins, at the growth cone. Activation of these receptor signals, called 'guidance cues', navigates the outgrowth of axons, playing a key role in brain development [4]. Several lines of evidence have shown that disruptions of these guidance cues lead to malformation of the corpus callosum and failure of neuronal projection [5].

Because the dysfunction of axon-guidance signaling is thought to be related to microstructural abnormalities in the brain of people with autism, as shown by neuroimaging studies, we examined the postmortem brains of people with autism to identify any changes in the expression of axon-guidance proteins.

## Methods

### Axon-guidance genes

We examined the postmortem brains of people with autism to identify any differential expressions of axon-guidance receptors, including (EFN)A4, eEFNB3, plexin (PLXN)A4, roundabout 2 (ROBO)2 and ROBO3. These candidate genes were selected because EFNA4, EFNB3 and PLXNA4 are located at autism-linked chromosome loci [6], ROBO2 expression was reported to be reduced in peripheral lymphocytes in people with autism [7], and, in a case-control genetic-association study, ROBO3 was linked with autism [7].

### Human postmortem brain samples

All experimental procedures were approved by the Institutional Review Board of Hamamatsu University School of Medicine. Tissue samples were obtained from the Autism Tissue Program (Princeton, NJ) and NICHD Brain and Tissue Bank for Developmental Disorders (Baltimore, MD).

The samples obtained were frozen postmortem samples of human left ACC (Brodmann area 24, ventral region), a region implicated in the pathophysiology of autism (that is,, the functional alteration of the region is correlated with severity of autistic symptoms) [8-10], taken from people with autism (n = 8) and age-matched controls (n = 13), and frozen postmortem samples of human primary motor cortex (PMC) (Brodmann area 4), which has been shown to exhibit no functional difference between people with autism and controls, taken from people with autism (n = 7) and age-matched controls (n = 8). The autism and control samples were further matched for gender and the postmortem interval (PMI) (Table 1). The diagnosis of autism was made according to the *Diagnostic and Statistical Manual*, Fourth Edition, Text Revision, and confirmed by the Autism Diagnostic Interview-Revised.

**Table 1:** Summary of demographic variables on brain samples

Variable	Control, n = 13	Autism, n = 8	*P*-value
Age, years^a^	15.6 ± 6.5 (8 to 28)	16.4 ± 8.3 (8 to 29)	0.818^b^
Gender, *n *(%)			1.0^c^
Male	9 (69)	6 (75)	
Female	4 (31)	2 (25)	
PMI, hours^a, d^	15.0 ± 5.6 (5 to 22)	18.0 ± 14.5 (9 to 50)	0.509^b^

^a^Mean ± SD (range)

^b^Derived from two-tailed unpaired *t*-test.

^c^Derived from Fisher's exact test.

^d^PMI, postmortem interval.

### Real-time quantitative reverse-transcriptase polymerase chain reaction

Differential expression of candidate genes was investigated by real-time reverse-transcriptase (RT)-PCR as described elsewhere [11], carried out in a fast real-time PCR system (ABI PRISM 7900HT; Applied Biosystems, Foster City, CA, USA) using Taqman probe detection, following the manufacturer's recommendations.

For the analysis of plexin A4, two isoforms (plexin A4A and plexin A4B) were tested. The expression of either gene relative to each of three internal control housekeeping genes (ß-actin, glyceraldehyde-3-phosphate dehydrogenase (GAPDH) and ß2-microglobin) was determined as 2^-ΔCt^, and normalized by the mean value of the controls.

### Western blotting

The protein levels of differentially expressed genes were further analyzed by western blotting as described previously [12]. Briefly, 40 μg of each brain sample was loaded onto a 7.5% SDS polyacrylamide gel and separated by electrophoresis. The proteins were electroblotted onto a polyvinylidene fluoride membrane (Millipore, Billerica, MA, USA), then blocked and incubated with the primary antibody at 4°C overnight, followed by incubation with a horseradish peroxidase-conjugated secondary antibody. The primary antibodies used were anti-ROBO2 (1:1000; R&D Systems, Minneapolis, MN, USA), anti-EFNB3 (1:500; R&D Systems), anti-PLXNA4 (1:500; Abcam, Cambridge, Cambridgeshire, UK) and anti-ß-actin (1:2000; Abcam). The densities of the immunoreactive bands were quantified and analyzed using Image-J software http://rsbweb.nih.gov/ij/.

### Statistical analysis

For the real-time PCR analysis, the resultant data had a non-normal distribution, thus the statistical analysis was conducted using the Mann-Whitney *U*-test. Because of the complexity of using three housekeeping genes as a reference, we considered a finding to be significant when the relative expression of the relevant candidate gene was significantly different at the *P *< 0.05 level from all three housekeeping genes. Thus, the significant results in our real-time RT-PCR analyses can be viewed as conservative.

For western blotting, he protein expression level of each enzyme was normalized by the level of ß-actin. The two-tailed unpaired *t*-test was used, and values of *P *< 0.05 were considered significant.

## Results

There was no significant difference in age (*t *= 0.16, degrees of freedom (d.f.) = 19, *P *= 0.88; *t *= 0.39, d.f. = 13, *P *= 0.70), PMI (*t *= 0.67, d.f. = 18, *P *= 0.51; *t *= 0.13, d.f. = 12, *P *= 0.90) or gender (*P = *1.0; *P = *1.0, Fisher's exact test) between the autistic and control groups (ACC and PMC, respectively).

**Figure 1:**
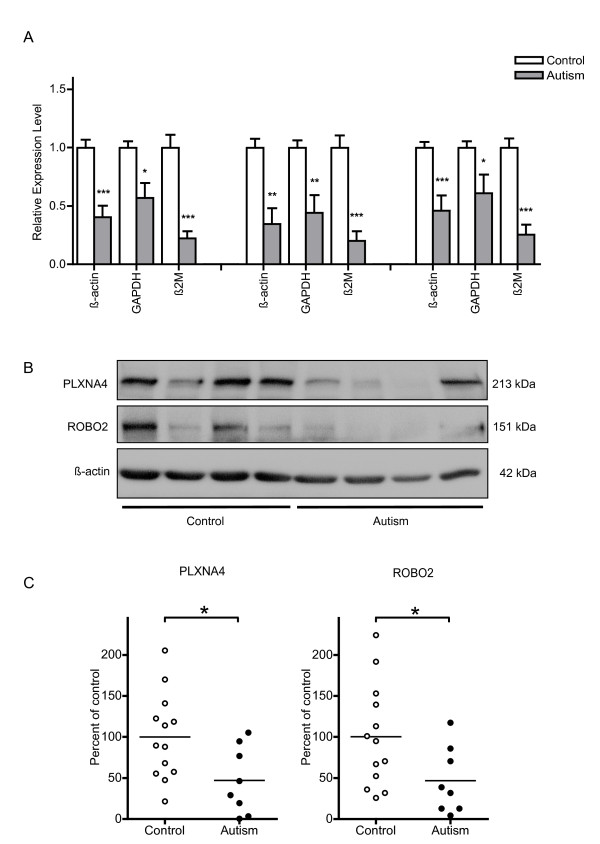
**Differential mRNA and protein expression of each candidate molecule**. **(A) **Differential mRNA expression of EFNB3, PLXNA4A and ROBO2 in the anterior cingulate cortex (ACC) of brains from people with autism and controls. Graphs represent the mean ratio (autism:control) of relative expression levels. The error bar represents the SEM. There were thirteen control and eight autistic samples. *P < 0.05, **P < 0.01, ***P < 0.001 by Mann-Whitney *U*-test. Left to right, *U *= 4, *P *< 0.001; *U *= 18, *P *= 0.015; *U *= 0., *P *< 0.001; *U *= 11, *P *< 0.003; *U *= 14, *P *< 0.007; *U *= 3, *P *< 0.001; *U *= 7, *P *< 0.001; *U *= 23, *P *< 0.039; *U *= 0., *P *< 0.001. **(B) **Western blots showing the immunolabeling of PLXNA4 and ROBO2 in the ACC of control and autistic samples. **(C) **The values in the graphs are the mean percentage of the mean of the control values. There were thirteen control and eight autistic samples. *P < 0.05 by two-tailed unpaired *t*-test. Left to right, *t *= 2.44, df = 19, *P *< 0.024; *t *= 2.14, df = 19, *P *< 0.045.

Real-time RT-PCR revealed that the relative expression levels of EFNB3, PLXNA4A and ROBO2, compared, with all three housekeeping genes were significantly lower in the ACC of people with autism than in the ACC of controls. The other axon-guidance signaling-related genes we examined (EFNA4, PLXNA4B and ROBO3), did not meet our stringent criteria for significance (Figure 1A). In addition, none of the genes tested in the study had significantly different expression in the PMC of autistic brains compared with the PMC of control brains.

The protein levels of the three genes that were shown by real-time RT-PCR to be differentially expressed in the ACC of autistic brains (that is,, EFNB3, PLXNA4A and ROBO2) were further examined using western blotting. The immunoreactive levels of PLXNA4 and ROBO2 were significantly reduced in the ACC of the autistic compared with that of the control brains, with a 53.0% reduction for PLXNA4 (*t *= -2.44, d.f. = 19, *P *< 0.05) and, a 53.4% reduction for ROBO2 (*t *= -2.14, d.f. = 19, *P *< 0.05) in the autism group relative to the control group (Figure 1B, C). However, the value of EFNB3 was not significantly different between the two groups (*t *= 1.43, *df *= 19, *P *= 0.17). We then examined the correlations between the mRNA/protein levels of the genes and the age, PMI or gender of the subjects, and found no significant difference.

## Discussion

In this study, we investigated the transcriptional and translational regulation of axon-guidance receptors in the ACC of people with autism. The results provide the first evidence, to our knowledge, of reduced mRNA and protein levels of axon-guidance receptors, including those of PLXNA4 and ROBO2, in the ACC of people with autism; this region has previously been implicated in the pathophysiology of autism. We found a downregulation of both types of receptors in the ACC, although such downregulation was not evident in the other region examined, the PMC.

PLXNA4 is a multiple semaphorin (Sema) receptor that mediates axon-repulsive signaling. Animal studies have shown that a disruption of PLXNA4/Sema signaling causes a malformation of white matter, such as the anterior commissure [13]. By contrast, ROBO2 is a receptor for Slit proteins, which are crucial for the proper development of forebrain connections. It is of particular interest that a loss of ROBO2/Slit function affects development of the serotonergic and dopaminergic systems [5], both of which are implicated in the pathophysiology of autism.

Our results regarding the altered regulation of axon-guidance receptors in the brains of autistic people, together with the finding that a loss of function of axon-guidance proteins elicits abnormalities of white-matter formation and axonal trajectory, are in line with findings from recent neuroimaging studies showing abnormalities of microstructure and neuronal connectivity in the brains of people with autism. For example, DTI studies showed reduced FA (that is,, impaired structural integrity) in white matter in the region of the ACC in people with autism [9]. However, to clarify the association between structural abnormalities and axon-guidance proteins in autism, further studies conducting both neuroimaging and relevant genetic analyses in the same individuals with the disorder will be necessary.

In addition, recent microarray analyses of a genetic mouse model for Smith-Lemli-Opitz Syndrome, which is characterized by mental retardation and multiple congenital anomalies with the behavioral features of autism, displayed dynamic gene-expression changes in axonal-guidance proteins in the brain [14]. Furthermore, recent studies have suggested positive associations of genes involved in the plexin/semaphorin signaling system and schizophrenia [15,16], which overlaps with autism in certain area of social dysfunction [17]. The findings of these studies, together with our results, suggest that abnormalities of axon-guidance proteins may be directly linked to the behavioral symptoms characteristic of autism.

Because the present study was limited by the small sample size, a larger sample size would bolster our findings. However, we used conservative statistical procedures to avoid type I error (three housekeeping genes were used as reference), along with western-blotting analysis to confirm the results. Therefore, our findings can be considered as evidence that axon-guidance proteins play an important role in the pathophysiology of autism.

## Competing interests

This work was free from any financial or other limitations that might have constituted a conflict of interest. In addition, none of the individual authors report any conflicts of interest.

## Authors' contributions

SS, KN, KK, HH and NM designed the research; SS, KI, CS, YK, AA, IT, HM, YI, KS and KK performed the research; SS, KJT, GS and NT analyzed data; and SS and NT wrote the paper. All authors read and approved the final manuscript.
